# Stochastic optimization on complex variables and pure-state quantum tomography

**DOI:** 10.1038/s41598-019-52289-0

**Published:** 2019-11-06

**Authors:** A. Utreras-Alarcón, M. Rivera-Tapia, S. Niklitschek, A. Delgado

**Affiliations:** 1 Instituto Milenio de Investigación en Óptica, Universidad de Concepción, Concepción, Chile; 2 Facultad de Ciencias Físicas y Matemáticas, Departamento de Física, Universidad de Concepción, Concepción, Chile; 3 Facultad de Ciencias Físicas y Matemáticas, Departamento de Estadística, Universidad de Concepción, Concepción, Chile

**Keywords:** Quantum information, Quantum mechanics, Quantum metrology

## Abstract

Real-valued functions of complex arguments violate the Cauchy-Riemann conditions and, consequently, do not have Taylor series expansion. Therefore, optimization methods based on derivatives cannot be directly applied to this class of functions. This is circumvented by mapping the problem to the field of the real numbers by considering real and imaginary parts of the complex arguments as the new independent variables. We introduce a stochastic optimization method that works within the field of the complex numbers. This has two advantages: Equations on complex arguments are simpler and easy to analyze and the use of the complex structure leads to performance improvements. The method produces a sequence of estimates that converges asymptotically in mean to the optimizer. Each estimate is generated by evaluating the target function at two different randomly chosen points. Thereby, the method allows the optimization of functions with unknown parameters. Furthermore, the method exhibits a large performance enhancement. This is demonstrated by comparing its performance with other algorithms in the case of quantum tomography of pure states. The method provides solutions which can be two orders of magnitude closer to the true minima or achieve similar results as other methods but with three orders of magnitude less resources.

## Introduction

Optimization plays an important role in quantum information theory. Quantum tomography of an unknown pure state |*ψ*〉 can be formulated as the minimization of the Infidelity $I=1-|\langle \psi |\varphi \rangle {|}^{2}$ with respect to $|\varphi \rangle$, where the minimizer is given by $|\varphi \rangle =|\psi \rangle$^1^. Similarly, the geometric measure of entanglement^2,3^ of a pure *n*-partite state |*ψ*〉 is defined as the minimum value of *I* with respect to the set of separable pure states^4^. Bell-like inequalities^5^ are functions of a quantum state, pure or mixed, and of measurement settings, typically observables. The highest violation for a fixed state is obtained by maximizing with respect to the set of observables to be measured.

The previous examples correspond to the optimization of real-valued target functions that are natively defined on the field of the complex numbers. Interestingly, this problem is still far from a complete understanding. Real-valued functions of complex variables do not satisfy the Cauchy-Riemann conditions, that is, there exists no Taylor series for this class of functions. Consequently, optimization with respect to complex variables usually requires to split these into their real and imaginary parts followed by an optimization with respect to real variables. This procedure has two unwanted effects. First, in the case of Taylor series based optimization methods, the gradient of the target function to be optimized is calculated with respect to the real and imaginary parts. The elements of this real-valued gradient are in general more convoluted than would be those of a complex gradient formed by first order derivatives with respect to the initial complex variables, as for instance, in Wirtinger calculus^6^. Second, any inherent structures present in the complex derivatives of the target function, which might be exploited to enhance the performance of optimization methods, are hidden. Thus, optimization methods designed to natively work on complex variables might lead to a performance improvement^7^. This is the case of neural networks, whose formulation on the field of complex numbers exhibits a performance boost^8,9^.

Here, we introduce the Complex simultaneous perturbation stochastic approximation (CSPSA), a numerical stochastic minimization method that can be directly applied to real-valued target functions of complex variables. These functions do not satisfy the Cauchy-Riemann conditions and, consequently, there exists no Taylor series for this class of functions.

The method is based on an estimation of the Wirtinger complex gradient of the target function, which is subsequently used to generate a sequence of complex estimates approaching the minimizer. Magnitude and direction of the gradient's estimate are calculated as the difference between the target function evaluated at two different points and as a complex vector whose components are randomly, independently generated, respectively. Thereby, all calculations are carried out within the field of complex numbers. The estimation of the complex gradient is asymptotically unbiased and the sequence of complex estimates converges to the solution of the minimization problem.

CSPSA enables the optimization of functions with unknown parameters, since the only input it requires are evaluations of the target function. For instance, in quantum tomography the value of the infidelity *I* can be obtained by measuring, on the system described by state |*ψ*〉, an observable that contains in its spectral decomposition the state $|\varphi \rangle$. Thus, we can obtain the values of *I* for any $|\varphi \rangle$ as long as |*ψ*〉 is an unknown but fixed parameter. Determining the amount of geometric entanglement^3,4^ of an unknown state is also within reach of CSPSA. In this case the infidelity of an unknown multipartite pure quantum state is minimized with respect to the set of separable states, which requires the measurement of local observables. The violation of the Claus-Horne-Shimony-Holt^5^ (CHSH) inequality with an unknown state, pure or mixed, can also be studied. In this case CSPSA maximizes the violation by driving the measurement bases to the optimal measurement setting.

CSPSA exhibits a large performance boost, in comparison to stochastic optimization algorithms for functions of real variables. We show this by applying CSPSA to the tomography of pure quantum states. Extensive numerical simulations via random sampling show that CSPSA achieves values of the mean infidelity orders of magnitude smaller than the ones provided by Self guided quantum tomography (SGQT), a quantum tomographic scheme based on a stochastic minimization method for functions of real variables^1^. These simulations consider the same amount of resources for both methods, that is, number of equally prepared quantum systems and total number of measurement outcomes or, equivalently, number of iterations and evaluations of the target function. Consequently, CSPSA leads to a considerable reduction in the resources required to estimate an unknown pure quantum state and provides a clear indication that optimization on complex variables can lead to higher performance methods. Furthermore, it has been shown that the use of resources by SGQT compares favorably to other quantum tomographic schemes^10^. Thus, CSPSA based quantum tomography provides a further improvement in the search for the efficient use of resources^11–16^ in the determination of quantum states.

## Method

The problem of optimizing a real-valued function of complex variables $f(z,{z}^{\ast }):{\mathfrak{C}}\to {\mathbb{R}}$, where the set ${\mathfrak{C}}$ is given by ${{\mathfrak{C}}}^{n}:\,=\{({\bf{z}},{{\bf{z}}}^{\ast })=({z}_{1},\ldots ,{z}_{n},{z}_{1}^{\ast },\ldots ,{z}_{n}^{\ast })\in {{\mathbb{C}}}^{n}\times {{\mathbb{C}}}^{n}\}$ with *n* = 1, can be completely stated within the field of complex numbers. This requires the definition of Wirtinger derivatives^6^ ${\partial }_{z}f=\frac{1}{2}({\partial }_{x}f-i{\partial }_{y}f)$ and ${\partial }_{{z}^{\ast }}f=\frac{1}{2}({\partial }_{x}f+i{\partial }_{y}f)$, where $z=x+iy\in {\mathbb{C}}$ and $x,y\in {\mathbb{R}}$. The Cauchy-Riemann equations establish necessary and sufficient conditions for the existence of the complex derivative $f^{\prime} (z)={\mathrm{lim}}_{\Delta z\to 0}\,[f(z+\Delta z)-f(z)]/\Delta z$ with $f,z\in {\mathbb{C}}$. Given the function *f*(*z*) = *u*(*x*, *y*) + *iv*(*x*, *y*) of $z=x+iy\in {\mathbb{C}}$ with *x*, *y*, *u* and $v\in {\mathbb{R}}$, the Cauchy-Riemann conditions are ∂_*x*_*u* = ∂_*y*_*v* and ∂_*y*_*u* = −∂_*x*_*v*. Thus, in terms of the Wirtinger derivatives, the Cauchy-Riemann conditions are equivalent to ${\partial }_{{z}^{\ast }}f=0$, in which case the (standard) complex derivative *f*′(*z*) agrees with the definition of ∂_*z*_*f*. However, Wirtinger derivatives ∂_*z*_*f* and ${\partial }_{{z}^{\ast }}f$ might exist even when the Cauchy-Riemann conditions do not hold. For example, for *f* = |*z*|^2^ we have ∂_*z*_*f* = z^*^ and ${\partial }_{{z}^{\ast }}f$ = *z*, while in this case the function *f* is non-holomorphic. Let us note that one of the advantages of Wirtinger derivatives is that they can be manipulated as real partial derivatives, where *z* and z^*^ are treated as independent variables since ∂_*z*_z^*^ = 0 = ∂_z*_z.

The search for stationary points of real-valued functions of complex variables cannot be carried out with the help of the standard complex derivative, which in this case does not exist. Therefore, the problem is studied at the level of the field of the real numbers by calculating the points at which the real gradient vanishes. Nevertheless, it is possible to define a complex vector gradient operator which allows for the search of stationary points easily and with mathematical rigor. For a complex-valued function *f*(***μ***) with ${\boldsymbol{\mu }}=({\bf{z}},{{\bf{z}}}^{\ast })\in {{\mathfrak{C}}}^{n}$ and an infinitesimal change *δ****μ*** = (*δ****z***, *δ*z^*^), the change *δf* in the value of the function *f* is given by^17^

1 $$\delta f=({\partial }_{{\boldsymbol{\mu }}}f)\delta {{\boldsymbol{\mu }}}^{t},$$

with the complex-valued gradient operator ∂_***μ***_ = (∂_***z***_, ∂_z*_) = (∂_*z*1_, …, ∂_*zn*_, ∂_*z*1*_, …, ∂_zn*_). In the case of a real-valued function *f*, we have that

2 $$\delta f=2\Re [({\partial }_{{{\bf{z}}}^{\ast }}f)\delta {{\bf{z}}}^{t}\mathrm{].}$$

Thereby, stationary points are completely characterized by the vanishing of the gradient ${\partial }_{{z}^{\ast }}f$= **0** or, equivalently, by ∂_***z***_*f* = 0^18,19^. Furthermore, for a given magnitude of *δ****z***, the maximum increase in *f* arises when *δ****z*** is in the direction of ${\partial }_{{z}^{\ast }}f$. This approach to the optimization of functions of complex variables, holomorphic or not, allows to keep all manipulations within the field of complex numbers as well as to obtain simpler expressions.

The Complex simultaneous perturbation stochastic approximation generates a sequence of estimates ${\hat{{\bf{z}}}}_{k}$ of the minimizer $\tilde{{\bf{z}}}$ of $f({\bf{z}},{{\bf{z}}}^{\ast }):\,{{\mathfrak{C}}}^{n}\to {\mathbb{R}}$, that is, ${\bf{g}}(\tilde{{\bf{z}}},{\tilde{{\bf{z}}}}^{\ast })={\partial }_{{{\bf{z}}}^{\ast }}f(\tilde{{\bf{z}}},{\tilde{{\bf{z}}}}^{\ast })=0$. The estimate ${\hat{{\bf{z}}}}_{k}$ of $\tilde{{\bf{z}}}$ at the k-th iteration is updated according to the iterative rule

3 $${\hat{{\bf{z}}}}_{k+1}={\hat{{\bf{z}}}}_{k}-{a}_{k}{\hat{{\bf{g}}}}_{k}({\hat{{\bf{z}}}}_{k},{\hat{{\bf{z}}}}_{k}^{\ast }),$$

with *a*_*k*_ a positive gain coefficient. Equation (3) resembles the Gradient (or Steepest) descent method, an iterative optimization algorithm for real functions of real variables that takes steps proportional to the negative direction of the real-valued gradient. Instead, CSPSA is based on an estimation ${\hat{{\bf{g}}}}_{k}$ of the gradient ***g*** of *f* with respect to z^*^. The i-th component ${\hat{g}}_{k,i}$ of ${\hat{{\bf{g}}}}_{k}$ is calculated as

4 $${\hat{g}}_{k,i}=\frac{f({\hat{{\bf{z}}}}_{k+},{\hat{{\bf{z}}}}_{k+}^{\ast })+{\varepsilon }_{k,+}-(f({\hat{{\bf{z}}}}_{k-},{\hat{{\bf{z}}}}_{k-}^{\ast })+{\varepsilon }_{k,-})}{2{c}_{k}{\Delta }_{k,i}^{\ast }},$$

with ${\hat{{\bf{z}}}}_{k\pm }={\hat{{\bf{z}}}}_{k}\pm {c}_{k}{{\boldsymbol{\Delta }}}_{k}$ and *c*_*k*_ a positive gain coefficient. The vector ${{\boldsymbol{\Delta }}}_{k}\in {{\mathbb{C}}}^{n}$ is randomly generated and ${\varepsilon }_{k,\pm }$ describe the presence of noise in the values of $f({\hat{{\bf{z}}}}_{k\pm },{\hat{{\bf{z}}}}_{k\pm }^{\ast })$.

The estimation of ***g*** by means of evaluations of *f* becomes an advantage when ***g*** is not readily available. For instance, the evaluation of ***g*** is computationally resource intensive, ***g*** cannot be directly inferred from measurements in real-time applications, the exact functional relationship between *f* and ***z*** is unknown, or *f* depends on a set of unknown parameters. The estimation ***g*^**_*k*_ requires the evaluation of *f* at two different vectors ${\hat{{\bf{z}}}}_{k\pm }$ regardless of the underlying dimension of the optimization problem. These evaluations are carried out by simultaneously varying all components of the vector ${\hat{{\bf{z}}}}_{k}$ through the addition and subtraction of the randomly generated components of the vector **Δ**_*k*_. CSPSA also allows for the presence of noise in the evaluations of *f*, which might occur due to experimental inaccuracies in the acquisition of the values of *f* or due to finite sample size effects. Other optimization methods have similar properties, for instance Simultaneous perturbation methods (SPM)^20^ and the Finite difference stochastic approximation (FDSA)^21^, which unlike CSPSA work on the field of the real numbers. SPM and FDSA are employed to optimize real-valued functions *f*(***x***) with ${\bf{x}}\in {{\mathbb{R}}}^{n}$ and are based on the update rule ***x***_*k*+1_ = ***x***_*k*_ − *a*_*k*_ ***g*^**_*k*_(***x***_*k*_), where ***g*^**_*k*_(***x***_*k*_) is an estimation of the real-valued gradient ▽_***x***_*f*(***x***_*k*_). This estimation is calculated on a point ***x***_*k*_, which is generated by means of a stochastic process. However, CSPSA maintains all calculations and updates of ${\hat{{\bf{z}}}}_{k}$, *f* and ***g*^**_*k*_ within the field of complex numbers.

Stochastic optimization algorithms, such as SPM and FDSA, which are characterized by an iterative rule as in Eq. (3) but on the field of the real numbers, have been intensively studied^22,23^ and conditions to guarantee local convergence have been firmly established. This can be suitably extended to encompass optimization on the field of the complex numbers by means of CSPSA. This is introduced in detail in the Supplementary Information by means of two theorems. In particular, it is possible to show that the sequence ${\hat{{\bf{z}}}}_{k}-\tilde{{\bf{z}}}$ as well as the conditional mean ${\mathbb{E}}[{\hat{{\boldsymbol{g}}}}_{k}{(}{\hat{{\boldsymbol{z}}}}_{k}{)}-{\boldsymbol{g}}{(}{\hat{{\boldsymbol{z}}}}_{k}{)}{|}{\hat{{\boldsymbol{z}}}}_{k}]$ vanish asymptotically. Thereby, the sequence of estimates ${\hat{{\bf{z}}}}_{k}$ provided by Eq. (3) converges almost surely to the minimizer $\tilde{{\bf{z}}}$ of the optimization problem and ***g*^**_*k*_ defined by Eq. (4) is an asymptotically unbiased estimation of the gradient ***g*** of *f*. A property is satisfied almost surely if it is satisfied with probability one. Equivalently, the property does not hold for a null measure set.

Convergence and unbiasedness of CSPSA require conditions on **Δ**_*k*_, *a*_*k*_, *c*_*k*_ and *f* that can be fulfilled with particular choices. The components of **Δ**_*k*_ are independent and identically generated by selecting at each iteration with equal probability values in the set $\{{e}^{2i{\nu }_{p}}\}$ with *p* = 0, …, *K* such that ${\sum }_{p}{e}^{2i{\nu }_{p}}\mathrm{=0}$. There is still, however, a considerable freedom in the choice of **Δ**_*k*_ which also allows for improving the rate of convergence. Our choice of **Δ**_*k*_ is given by *ν*_*p*_ = {0, *π*/4, *π*/2, 3*π*/4}. This corresponds to a vector in ${{\mathbb{R}}}^{2n}$ with vanishing components, which does not satisfy the conditions for the convergence of SPM and thus it cannot be employed as the direction of the estimation of a real gradient. The gain coefficients *a*_*k*_ and *c*_*k*_ control the convergence of CSPSA and are chosen as

5 $${a}_{k}=\frac{a}{{(k+1+A)}^{s}},\,{c}_{k}=\frac{b}{{(k+1)}^{r}}.$$

This choice is also employed in SPM. The values of *a*, *A*, *s*, *b* and *r* are adjusted to optimize the rate of convergence and depend on the target function. These are chosen in the case of CSPSA as the values which optimize the convergence of SPM in the asymptotic regime, that is, for a large number of iterations. Interestingly, these values lead to a much higher rate of convergence of CSPSA in the regime of a few iterations, when compared to SPM with standard (*s* = 0.602, *r* = 0.101, *A* = 0, *a* = 3, *b* = 0.1) or asymptotic (*s* = 1, *r* = 0.166, *A* = 0, *a* = 3, *b* = 0.1) gains. In the case of SPM, standard gains provide in the regime of a small number of iterations a faster convergence than the asymptotic gains.

An unknown pure quantum state |*ψ*〉 can be completely determined by minimizing the infidelity $I({\bf{z}})=1-|\langle \psi |\varphi ({\bf{z}})\rangle {|}^{2}$ with respect to the complex variables *z*_*i*_ that define the known pure quantum state $|\varphi ({\bf{z}})\rangle ={\sum }_{i}{z}_{i}|i\rangle /\sqrt{{\sum }_{i}|{z}_{i}{|}^{2}}$. The complex coefficients of state |*ψ*〉 entering in *I*(***z***) are considered to be unknown but fix parameters and the global minimum *I* = 0 is achieved when $|\varphi \rangle$ = |*ψ*〉, for any |*ψ*〉. The optimization of *I*(***z***) by means of CSPSA, Eqs (3) and (4), requires at each iteration the values *I*(***z***_*k*,±_), which are experimentally obtained by projecting the system in the unknown state |*ψ*〉 onto a base containing the state $|\varphi ({{\bf{z}}}_{k,\pm })\rangle$. The values *I*(***z***_*k*,±_) are then estimated as 1 − *n*_*k*,±_/*N* where *n*_*k*,±_ is the number of times the state $|\varphi ({{\bf{z}}}_{k,\pm })\rangle$ is detected and *N* is the total number of detections. Thereby, the total number of available copies *N*_*tot*_ of the quantum system in the unknown state is distributed among the total number of experiments for estimating two values of *I* at each iteration and the total number of iterations *k*, that is, *N*_*tot*_ = 2*Nk*. Noise tolerance of CSPSA guaranties convergence even when projecting onto states slightly different than $|\varphi ({{\bf{z}}}_{k,\pm })\rangle$.

The optimization of the infidelity can also be carried out on the field of the real numbers. In this case, the components of ***z*** are mapped onto the real numbers with the help of polar angles entering in hyper-spherical coordinates and arguments of complex phases. The infidelity becomes *I*(***x***) and now is possible to apply an optimization algorithm in the SPM family, for instance the Simultaneous perturbation stochastic approximation (SPSA)^24^. This employs an estimation of the real gradient and is described by Eqs (3) and (4) but replacing the complex vector ***z***_*k*_ by the real vector ***x***_*k*_. The components of **Δ**_*k*_ are independently and identically distributed and randomly selected from the set {+1, −1}. The application of SPSA to the determination of pure states has been introduced in the literature as SGQT and experimentally demonstrated^10^. Since CSPSA and SPSA (or SGQT) require at each iteration of exactly the same number and type of measurements, they are a perfect match for a comparative performance analysis.

## Results

**Figure 1:**
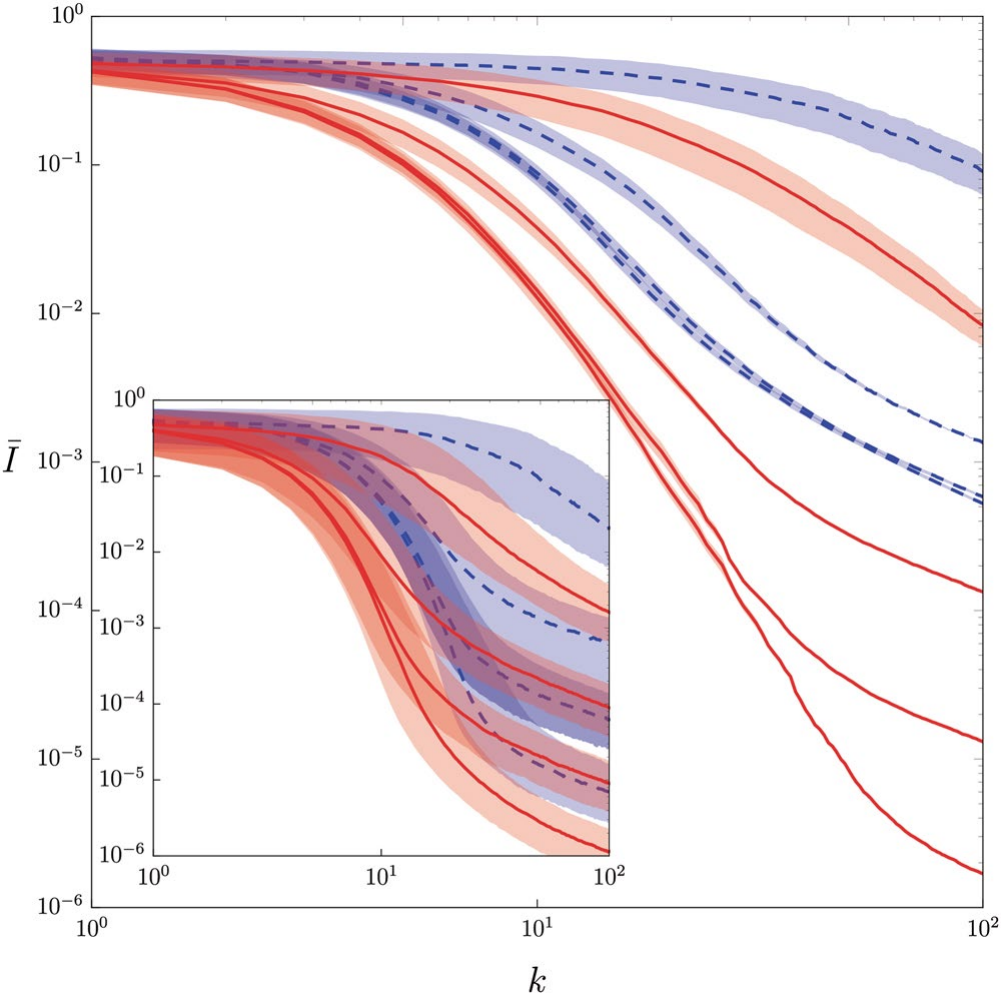
Mean infidelity *Ī*, averaged over the Hilbert space with 10^4^ pairs of unknown state and initial guess state, as function of the number *k* of iterations for single qubit quantum tomography via CSPSA (red continuous line) and SPSA (blue dashed line). Shaded areas indicate variance around mean. Inset exhibits median and interquartile range. From top to bottom red (blue) lines for *N* = 10, 10^2^, 10^3^ and 10^4^. For CSPSA *s* = 1 and *r* = 0.166 and for SPSA *s* = 0.602 and *r* = 0.101. For both methods *A* = 0, *a* = 3 and *b* = 0.1.

**Figure 2:**
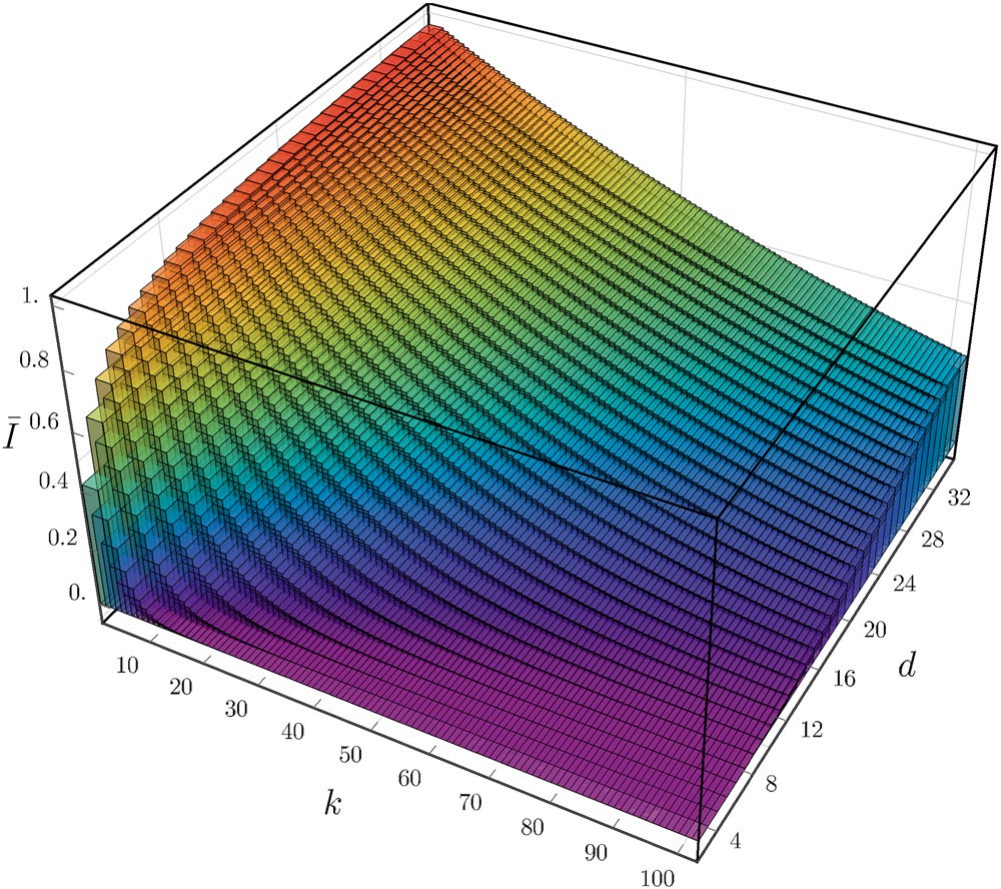
Mean infidelity *Ī*, calculated over 10^4^ realizations, as function of the dimension *d* of the Hilbert space and the number of iterations *k* for an ensemble size *N* = 10^3^. Other values as in Fig. 1.

Figure 1 shows the mean infidelity *Ī*, obtained by sampling according to the Haar distribution an ensemble of 10^4^ pairs of unknown states and initial guess states, as a function of *N* and *k* for the quantum tomography of a single qubit via CSPSA and SPSA. CSPSA achieves for *k* = 100 a mean infidelity which is at least 1 order of magnitude smaller than SPSA for a fixed amount of resources *N*_*tot*_. Thus, CSPSA clearly leads to an enhancement of the performance. The best mean infidelity achieved by SPSA at *k* = 100 is *Ī* ≈ 5 × 10^−4^ with *N* = 10^4^, that is, *N*_*tot*_ = 2 × 10^6^. This mean infidelity value can be achieved by CSPSA at *k* = 40 with *N* = 10^2^, that is, *N*_*tot*_ = 8 × 10^3^. Thereby, CSPSA offers a performance comparable to SPSA but with a large reduction in the amount of resources. The inset in Fig. 1 reproduces our performance analysis by means of the median and the interquartile range for both methods, where CSPSA still exhibits a performance boost over SPSA. At this point we note that there is no known proof for the convergence of the median for SPSA or CSPSA. In the case of CSPSA median and mean infidelity exhibit close values while SPSA shows a large difference between these figures. This is an indication that SPSA produces an asymmetric distribution for the infidelity which is much wider than the one generated by CSPSA. Figure 2 shows the mean infidelity generated by CSPSA as function of the number of iterations and the dimension. To achieve a predefined mean infidelity the number of required iterations increases with the dimension. Numerical simulations indicate that in the regime of a small number of iterations, that is, *k* ≤ 100, and for the inspected dimensions, that is, *d* ≤ 32, CSPSA surpasses SPSA, both in mean and in median.

CSPSA has other feasible applications to target functions with unknown parameters. For instance, the geometric measure of entanglement^2^ of a pure *n*-partite state |*ψ*〉 defined as ${E}_{{\sin }^{2}}={{\rm{\min }}}_{|\varphi \rangle }\mathrm{(1}-|\langle \psi |\varphi \rangle {|}^{2})$^4^, where the optimization is carried out onto the set of separable states $|\varphi \rangle =|\varphi ({{\bf{z}}}_{1}){\rangle }_{1}|\varphi ({{\bf{z}}}_{2}){\rangle }_{2}\ldots |\varphi ({{\bf{z}}}_{n}){\rangle }_{n}$. CSPSA can be employed, likewise quantum tomography via the optimization of *I*, to obtain the value of ${E}_{{sin}^{2}}$ for an unknown pure *n*-partite state by independently varying the local variables ***z***_*i*_. Violation of Bell-like inequalities^5^ also provide an interesting application of CSPSA. These are functions of a quantum state *ρ*, pure or mixed, and of measurement settings, typically observables. The maximal violation is obtained by optimizing with respect to the observables to be measured, which assumes the state *ρ* is known. If this is not the case, then we can apply CSPSA to the bases defining the observables in order to optimize the violation of the inequality. Thereby, the measurement of entanglement and the violation of Bell-like inequalities with unknown states can be implemented with the help of local measurements driven by CSPSA. The determination of ground state energy of complex physical systems^25^ and the post-processing of quantum tomographic data via maximum-likelihood estimation^26–28^ are difficult optimization problems due the large number of variables involved. Since CSPSA requires two evaluations of the target function independently of the number of complex variables, these problems might benefit from CSPSA. The utility of this methods goes beyond quantum mechanic and quantum information theory. Radio interferometric gain calibration^29^ is naturally stated as a non-linear least squares optimization problem onto the complex field. Here, for an interferometer array of antennas the measured pairwise visibilities ${\tilde{d}}_{p,q}$ between antennas *p* and *q* are employed to estimate the values of the complex gains *g*_*p*_ entering in the model ${d}_{p,q}={g}_{p}{m}_{p,q}{g}_{q}^{\ast }$ by optimizing the quantity ${\sum }_{p,q}|{d}_{p,q}-{\tilde{d}}_{p,q}{|}^{2}$ with respect to the set of gains^30^, where where *m*_*p*,*q*_ is the sky coherency. Other problems formulated in terms of optimization on the field of complex numbers are Coherent diffractive imaging^31^ and Multiple-input Multiple-out systems^32^.

## Discussion

In summary, CSPSA allows to optimize real-valued functions of complex variables. This makes unnecessary to recast the problem as the minimization of a more convoluted function of real variables. CSPSA shares several properties with the family of SPM: no need to evaluate the gradient of the target function, a reduced number of evaluations of the target function, noise tolerance, asymptotic unbiasedness and convergence in mean to the minimizer. However, CSPSA can achieve a large performance enhancement when compared with methods within this family, as for instance SPSA. We show this at hand of an important problem: Tomography of pure quantum states. Here, CSPSA outperforms SPSA when employing the same resources, or provides a similar performance but with far less resources. Thus, CSPSA constitutes a clear indication that optimization methods formulated within the field of complex numbers can lead to higher performances and provides a guideline for generalizing other optimization methods to the field of complex numbers, such as for instance preconditioned gradient methods^33^. There are several scenarios where the performance of quantum tomography via CSPSA can be enhanced. For instance, CSPSA requires two values of the Infidelity at each iteration. These are obtained by projecting onto two orthonormal bases, which generates 2*d* − 2 probabilities. Only two of them are employed by CSPSA. It is thus possible that the concatenation of CSPSA to an inference method, such as maximum likelihood estimation or bayesian inference, leads to a further speed up of the convergence of the tomographic method. This a very interesting possibility. As Fig. (1) suggests, the mean Infidelity provided by CSPSA seems to enter into an asymptotic regime, that is, *Ī* ≈ *α*(*d*)/*N*, where *α*(*d*) is a function of the dimension *d*. A suitable choice of the inference method might lead to *α*(*d*) ≈ *d* − 1. Thereby, the tomographic method would reach the Gill-Massar lower bound for the estimation accuracy of pure states^34–38^. We have based the tomographic method on the measurement of the Infidelity. It is, however, possible to employ other metrics, such as, for instance, mean squared error, that can be measured in interferometric experiments. We can also consider an extension of the present results to the case of reconstructing unknown coherent states and Schrödinger cat states of the electromagnetic field, where the Infidelity can be measured as the probability of projecting a displaced coherent state onto the vacuum state. Finally, we mention that an experimental demonstration of CSPSA in higher dimensions is within reach of current experimental setups^11,39–41^ based on single photons and concatenated spatial light modulators.

## Supplementary information

Supplementary
