# Supplementary material for: Stochastic optimization on complex variables and pure-state quantum tomography
Source: Sci Rep. 2019 Nov 6;9:16143. doi: 10.1038/s41598-019-52289-0 (PMC6834649; doi:10.1038/s41598-019-52289-0)
Supplement: Supplementary file 1 — Supplementary [file 41598_2019_52289_MOESM1_ESM.pdf]

Stochastic optimization on complex variables and pure-state quantum tomography
Supplementary Information

A. Utreras-Alarcón,[1,2] M. Rivera-Tapia,[1,2] S. Niklitschek,[3] and A. Delgado[1,2]

[1]*Instituto Milenio de Investigación en Óptica, Universidad de Concepción, Concepción, Chile*

[2]*Facultad de Ciencias Físicas y Matemáticas, Departamento de Física, Universidad de Concepción, Concepción, Chile*

[3]*Facultad de Ciencias Físicas y Matemáticas, Departamento de Estadística, Universidad de Concepción, Concepción, Chile*

We define the bias term for the estimator for the gradient at the $k-$th iteration of CSPSA as

$$\boldsymbol{b}_k(\hat{\boldsymbol{\mu}}_k) = \mathbb{E}(\hat{\boldsymbol{g}}_k(\hat{\boldsymbol{\mu}}_k) - \boldsymbol{g}(\hat{\boldsymbol{\mu}}_k)|\hat{\boldsymbol{\mu}}_k), \tag{1}$$

and the error term for the k-th iteration of CSPSA as

$$\boldsymbol{e}_k(\hat{\boldsymbol{\mu}}_k) = \hat{\boldsymbol{g}}_k(\hat{\boldsymbol{\mu}}_k) - \mathbb{E}(\hat{\boldsymbol{g}}_k(\hat{\boldsymbol{\mu}}_k)|\hat{\boldsymbol{\mu}}_k). \tag{2}$$

We also define the set $\mathscr{F}_k = \sigma(\{\hat{\boldsymbol{\mu}}_1, \hat{\boldsymbol{\mu}}_2, \cdots, \hat{\boldsymbol{\mu}}_k\})$. Because the $\boldsymbol{\Delta}_k$ are independent of $\mathscr{F}_k$, we have that

$$\mathbb{E}(\hat{\boldsymbol{g}}_k(\hat{\boldsymbol{\mu}}_k)|\mathscr{F}_k) = \mathbb{E}(\hat{\boldsymbol{g}}_k(\hat{\boldsymbol{\mu}}_k)|\hat{\boldsymbol{\mu}}_k). \tag{3}$$

Let us now consider the real vector $\boldsymbol{x} = (\mathrm{Re}(\boldsymbol{z}), \mathrm{Im}(\boldsymbol{z}))$. There is an isomorphism between the space of vectors $\boldsymbol{x}$ and the space of vectors $\boldsymbol{\mu} = (\boldsymbol{z}, \boldsymbol{z}^*)$. Indeed, the matrix

$$J = \begin{bmatrix} \mathbb{I}_n & i\mathbb{I}_n \\ \mathbb{I}_n & -i\mathbb{I}_n \end{bmatrix} \tag{4}$$

satisfies $\boldsymbol{\mu} = J\boldsymbol{x}$ and is invertible. Therefore, we can regard any function of $\boldsymbol{\mu}$ as a function of $\boldsymbol{x}$ [1].

Lemma 1. *Let W be a vector space on $\mathbb{R}$, and $U \subseteq \mathfrak{C}^n$ an open subset. Let $f : U \to W$ such that $f \in \mathscr{C}^K$, $K \geq 1$. Let us choose $\boldsymbol{\mu}_0 \in U$ and $r > 0$ such that $B_r(\boldsymbol{\mu}_0) \subset U$. Let us choose $\boldsymbol{h} \in \mathfrak{C}^n$ such that $\|h\| < r$. For positive integers $j < K$, we define the differential operator $\mathscr{D}_{\boldsymbol{h}}^{(j)}$ over f as*

$$(\mathscr{D}_{\boldsymbol{h}}^{(j)} f)(\boldsymbol{\mu}_0) = \sum_{i_1,\ldots,i_j} h_1 \cdot \ldots \cdot h_j \frac{\partial^j f}{\partial \mu_{i_1} \ldots \partial \mu_{i_j}}(\boldsymbol{\mu}_0). \tag{5}$$

Then,

$$f(\boldsymbol{\mu}_0 + \boldsymbol{h}) = \sum_{j=0}^{2} \frac{1}{j!} (\mathscr{D}_{\boldsymbol{h}}^{(j)} f)(\boldsymbol{\mu}_0) + R_{2,\boldsymbol{h}}(\boldsymbol{\mu}_0), \tag{6}$$

where

$$R_{2,\boldsymbol{h}}(\boldsymbol{\mu}_0) = \int_0^1 (1-t)[(\mathscr{D}_{\boldsymbol{h}}^{(2)} f)(\boldsymbol{\mu}_0 + t\boldsymbol{h}) - (\mathscr{D}_{\boldsymbol{h}}^{(2)} f)(\boldsymbol{\mu}_0)]\mathrm{d}t, \tag{7}$$

Proof. Because we can regard a function of $\boldsymbol{\mu}$ as a function of $\boldsymbol{x}$, we have $f(\boldsymbol{\mu}_0 + \boldsymbol{h}) = f(\boldsymbol{x}_0 + \boldsymbol{k})$. From theorem 5.8 in [2], we have that

$$f(\boldsymbol{x}_0 + \boldsymbol{k}) = \sum_{j=0}^{2} \frac{1}{j!} (D_{\boldsymbol{k}}^{(j)} f)(\boldsymbol{x}_0) + P_{2,\boldsymbol{k}}(\boldsymbol{x}_0) \tag{8}$$

with

$$P_{2,k}(\boldsymbol{x_0}) = \int_0^1 (1-t)[(D_{\boldsymbol{k}}^{(2)} f)(\boldsymbol{x}_0 + t\boldsymbol{k}) - (D_{\boldsymbol{k}}^{(2)} f)(\boldsymbol{x}_0)] \tag{9}$$

where $\boldsymbol{x}_0 = J^{-1}\boldsymbol{\mu}_0$, $\boldsymbol{k} = J^{-1}\boldsymbol{h}$ and the real differential operator $D_{\boldsymbol{k}}^{(j)}$ onto function f is

$$(D_{\boldsymbol{k}}^{(j)} f)(\boldsymbol{x}) = \sum_{i_1,\cdots,i_j}^{2n} k_{i_1} \cdots k_{i_j} (\partial_{x_{i_1}} \cdots \partial_{x_{i_j}} f)(\boldsymbol{x}). \tag{10}$$

Then,

$$\begin{aligned} f(\boldsymbol{x}_0 + \boldsymbol{k}) &= f(\boldsymbol{x}_0) + \sum_{i=1} k_i \frac{\partial f}{\partial x_i}(\boldsymbol{x}_0) + \frac{1}{2}\sum_{i=1} k_i^2 \frac{\partial^2 f}{\partial {x_i}^2}(\boldsymbol{x}_0) \\ &+ \sum_{i<j} k_i k_j \frac{\partial^2 f}{\partial x_i \partial x_j}(\boldsymbol{x}_0) + P_{2,\boldsymbol{k}}(\boldsymbol{x}_0) \\ &= f(\boldsymbol{x}_0) + \boldsymbol{k}^T \frac{\partial f}{\partial \boldsymbol{x}}(\boldsymbol{x}_0) \\ &+ \frac{1}{2}\boldsymbol{k}^T \frac{\partial^2 f}{\partial \boldsymbol{x} \partial \boldsymbol{x}^T}(a)\boldsymbol{k} + P_{2,\boldsymbol{k}}(\boldsymbol{x}_0). \end{aligned}$$

Because we have $f(\boldsymbol{x}_0) = f(\boldsymbol{\mu}_0)$:

$$\begin{aligned} f(\boldsymbol{x}_0 + \boldsymbol{k}) &= f(\boldsymbol{\mu}_0) + \boldsymbol{h}^T (J^{-1})^T J^T \frac{\partial f}{\partial \boldsymbol{\mu}}(\boldsymbol{\mu}_0) \\ &+ \frac{1}{2}\boldsymbol{h}^T (J^{-1})^T J^T \frac{\partial^2 f}{\partial \boldsymbol{\mu} \partial \boldsymbol{\mu}^T}(\boldsymbol{\mu}_0) J J^{-1}\boldsymbol{h} + P_{2,\boldsymbol{k}}(\boldsymbol{x}_0) \\ &= f(\boldsymbol{\mu}_0) + \boldsymbol{h}^T \frac{\partial f}{\partial \boldsymbol{\mu}}(\boldsymbol{\mu}_0) \\ &+ \frac{1}{2}\boldsymbol{h}^T \frac{\partial^2 f}{\partial \boldsymbol{\mu} \partial \boldsymbol{\mu}^T}(\boldsymbol{\mu}_0)\boldsymbol{h} + P_{2,\boldsymbol{k}}(\boldsymbol{x}_0) \\ &= \sum_{j=0}^{2} \frac{1}{j!} (D_{\boldsymbol{h}}^{j} f)(\boldsymbol{\mu}_0) + P_{2,\boldsymbol{k}}(\boldsymbol{x}_0). \end{aligned}$$

Also,

$$
\begin{aligned}
P_{2,\boldsymbol{k}}(\boldsymbol{x}_0) &= \int_0^1 (1-t)((D^2_{\boldsymbol{k}}f)(\boldsymbol{x}_0+t\boldsymbol{k})-(D^2_{\boldsymbol{k}}f)(\boldsymbol{x}_0))\mathrm{d}t \\
&= 2\int_0^1 (1-t)\left[\frac{1}{2}\sum_{i=0} k_i^2\left(\frac{\partial^2 f}{\partial x_i^2}(\boldsymbol{x}_0+t\boldsymbol{k})-\frac{\partial^2 f}{\partial x_i^2}(\boldsymbol{x}_0)\right)+\sum_{i<j} h_{[R]j}h_{[R]j}\left(\frac{\partial^2 f}{\partial x_i \partial x_j}(\boldsymbol{x}_0+t\boldsymbol{k})-\frac{\partial^2 f}{\partial x_i \partial x_j}(\boldsymbol{x}_0)\right)\right]\mathrm{d}t \\
&= \int_0^1 (1-t)\boldsymbol{k}^T\left(\frac{\partial^2 f}{\partial \boldsymbol{x}\partial \boldsymbol{x}^T}(\boldsymbol{x}_0+t\boldsymbol{k})-\frac{\partial^2 f}{\partial \boldsymbol{x}\partial \boldsymbol{x}^T}(\boldsymbol{x}_0)\right)\boldsymbol{k}\mathrm{d}t \\
&= \int_0^1 (1-t)\boldsymbol{h}^T\left(\frac{\partial^2 f}{\partial \boldsymbol{\mu}\partial \boldsymbol{\mu}^T}(\boldsymbol{\mu}_0+t\boldsymbol{h})-\frac{\partial^2 f}{\partial \boldsymbol{\mu}\partial \boldsymbol{\mu}^T}(\boldsymbol{\mu}_0)\right)\boldsymbol{h}\mathrm{d}t \\
&= \int_0^1 (1-t)((D^2_{\boldsymbol{h}}f)(\boldsymbol{\mu}_0+t\boldsymbol{h})-(D^2_{\boldsymbol{h}}f)(\boldsymbol{\mu}_0))\mathrm{d}t \\
&= R_{2,\boldsymbol{h}}(\boldsymbol{\mu}_0).
\end{aligned}
$$

Therefore,

$$
f(\boldsymbol{\mu}_0+\boldsymbol{h}) = \sum_{j=0}^{2}\frac{1}{j!}(D^{(j)}_{\boldsymbol{h}}f)(\boldsymbol{\mu}_0)+R_{2,\boldsymbol{h}}(\boldsymbol{\mu}_0). \tag{11}
$$

□

Theorem 1. *For α_0, α_1 and α_2 positive real constants and $\Omega = \{\omega\}$ the sample space that generates the sequence $\hat{\boldsymbol{z}}_1, \hat{\boldsymbol{z}}_2, \ldots$, consider all $k \geq K$ for some $K < \infty$, and suppose that for each such k the $\Delta_{k,i}$ are independent and identically distributed and such that $\mathbb{E}(\Delta_{k,i}) = 0$, with $|\Delta_{k,i}| \leq \alpha_0$ a.s., $\mathbb{E}(|\Delta_{k,i}^{-1}|) \leq \alpha_1$ and $\mathbb{E}[e^{2i\phi_{k,i}}] = 0$, where $\phi_{k,i}$ is the phase of $\Delta_{k,i}$ in its polar decomposition. For example, $\Delta_{k,i}$ can be taken as symmetrically distributed about 0. For almost all $\hat{\boldsymbol{\mu}}_k$ (at each $k \geq K$) suppose that for all $\boldsymbol{\mu}$ in an open neighborhood of $\hat{\boldsymbol{\mu}}_k$, that is not an function of k or ω, $f \in \mathscr{C}^2(\mathfrak{C}^n)$ with $|\partial_{\mu_i}\partial_{\mu_j} f(\boldsymbol{\mu})| \leq \alpha_2$. Then for almost all $\omega \in \Omega$*

$$
\boldsymbol{b}_k(\hat{\boldsymbol{\mu}}_k) = \mathscr{O}(c_k). \tag{12}
$$

Proof. We apply lemma 1 to $f(\boldsymbol{\mu})$. Besides, because $|\partial_i \partial_j f(\boldsymbol{\mu})|$ is bounded, by hypothesis, $\mathbb{E}(|\partial_i \partial_j f(\boldsymbol{\mu})|)$ is well defined. For all $l \in \{1, \cdots, p\}$:

$$
b_{k,l}(\hat{\boldsymbol{\mu}}_k) = \mathbb{E}\left(\hat{g}_{k,l}(\hat{\boldsymbol{\mu}}_k)-g_l(\hat{\boldsymbol{\mu}}_k)\,|\hat{\boldsymbol{\mu}}_k\right) = \mathbb{E}\left(\left[\frac{f(\hat{\boldsymbol{z}}_k+c_k\boldsymbol{\Lambda}_k)-f(\hat{\boldsymbol{z}}_k-c_k\boldsymbol{\Lambda}_k)}{2c_k\Delta^*_{k,l}}-\frac{\partial f}{\partial z_l^*}(\hat{\boldsymbol{\mu}}_k)\right]\middle|\hat{\boldsymbol{\mu}}_k\right) \tag{13}
$$

where $\Lambda = (\Delta, \Delta^*)$.

When we expand $f(\hat{\boldsymbol{\mu}}_k \pm c_k\boldsymbol{\Lambda}_k)$, the terms with even powers of Λ_k will cancel each other.

$$
\begin{aligned}
b_{k,l}(\hat{\boldsymbol{\mu}}_k) &= \mathbb{E}\left(\left[2\sum_{i=1}^{2n}\frac{c_k\Lambda_{k,i}}{2c_k\Delta^*_{k,l}}\frac{\partial f}{\partial \mu_i}(\hat{\boldsymbol{\mu}}_k))\right.\right. \\
&\qquad \left.\left.+\frac{R_{2,c_k\boldsymbol{\Lambda}_k}(\hat{\boldsymbol{\mu}}_k)-R_{2,-c_k\boldsymbol{\Lambda}_k}(\hat{\boldsymbol{\mu}}_k)}{2c_k\Delta^*_{k,l}}-\frac{\partial f}{\partial z_l^*}(\hat{\boldsymbol{\mu}}_k)\right]\middle|\hat{\boldsymbol{\mu}}_k\right) \\
&= \mathbb{E}\left(\left[\sum_{i=1}^{n}\frac{\Delta_{k,i}}{\Delta^*_{k,l}}\frac{\partial f}{\partial z_i}(\hat{\boldsymbol{\mu}}_k)+\sum_{i\neq l}^{n}\frac{\Delta^*_{k,i}}{\Delta^*_{k,l}}\frac{\partial f}{\partial z_i^*}(\hat{\boldsymbol{\mu}}_k)\right.\right. \\
&\qquad \left.\left.+\frac{R_{2,c_k\boldsymbol{\Lambda}_k}(\hat{\boldsymbol{\mu}}_k)-R_{2,-c_k\boldsymbol{\Lambda}_k}(\hat{\boldsymbol{\mu}}_k)}{2c_k\Delta^*_{k,l}}\right]\middle|\hat{\boldsymbol{\mu}}_k\right).
\end{aligned}
$$

Because $\partial_i f(\hat{\boldsymbol{\mu}}_k)$ is completely determined by $\hat{\boldsymbol{\mu}}_k$,

$$
\mathbb{E}\left(\sum_{i\neq l}^{n}\frac{\Delta^*_{k,i}}{\Delta^*_{k,l}}\frac{\partial f}{\partial z_i^*}(\hat{\boldsymbol{\mu}}_k)\middle|\hat{\boldsymbol{\mu}}_k\right) = \mathbb{E}\left(\sum_{i\neq l}^{n}\frac{\Delta^*_{k,i}}{\Delta^*_{k,l}}\middle|\hat{\boldsymbol{\mu}}_k\right)\frac{\partial f}{\partial z_i^*}(\hat{\boldsymbol{\mu}}_k). \tag{14}
$$

Besides, the $\{\Delta_{k,i}\}$ are independently distributed, and are independent of $\hat{\boldsymbol{\mu}}_k$, so $\mathbb{E}\left(\sum_{i\neq l}^{n}\frac{\Delta^*_{k,i}}{\Delta^*_{k,l}}\middle|\hat{\boldsymbol{\mu}}_k\right) = \mathbb{E}((\Delta^*_{k,l})^{-1})\sum_{i\neq l}^{n}\mathbb{E}(\Delta^*_{k,i})$. Then

$$|b_{k,l}(\hat{\boldsymbol{\mu}}_k)| \leq \left|\mathbb{E}\left(\frac{\Delta_{k,l}}{\Delta_{k,l}^*}\right)\frac{\partial f}{\partial z_l^*}(\hat{\boldsymbol{\mu}}_k)\right| + \left|\mathbb{E}((\Delta_{k,l}^*)^{-1})\sum_{i\neq l}^{n}\left(\mathbb{E}(\Delta_{k,i})\frac{\partial f}{\partial z_i}(\hat{\boldsymbol{\mu}}_k)+\mathbb{E}(\Delta_{k,i}^*)\frac{\partial f}{\partial z_i^*}(\hat{\boldsymbol{\mu}}_k)\right)\right| + \left|\mathbb{E}\left(\frac{R_{2,c_k\boldsymbol{\Lambda}_k}(\hat{\boldsymbol{\mu}}_k)-R_{2,-c_k\boldsymbol{\Lambda}_k}(\hat{\boldsymbol{\mu}}_k)}{2c_k\Delta_{k,l}^*}\middle|\hat{\boldsymbol{\mu}}_k\right)\right|.$$

Because $\mathbb{E}(\Delta_{ik}) = \mathbb{E}(\Delta_{ik}^*) = 0$,

$$|b_{k,l}(\hat{\boldsymbol{\mu}}_k)| \leq \left|\mathbb{E}(e^{2i\phi_{kl}})\frac{\partial f}{\partial z_l^*}(\hat{\boldsymbol{\mu}}_k)\right| + \mathbb{E}\left(\frac{\left|R_{2,c_k\boldsymbol{\Delta}_k}(\hat{\boldsymbol{\mu}}_k)-R_{2,-c_k\boldsymbol{\Lambda}_k}(\hat{\boldsymbol{\mu}}_k)\right|}{\left|2c_k\Lambda_{k,l}^*\right|}\middle|\hat{\boldsymbol{\mu}}_k\right).$$

Therefore $\mathbb{E}(e^{2i\phi_{kl}}) = 0$,

$$|b_{k,l}(\hat{\boldsymbol{\mu}}_k)| \leq \mathbb{E}\left(\left|\int_0^1 \frac{1-t}{2c_k\Delta_{k,l}}\Big[(\mathscr{D}^2_{c_k\boldsymbol{\Lambda}_k}f)(\hat{\boldsymbol{\mu}}_k+tc_k\boldsymbol{\Lambda}_k) - (\mathscr{D}^2_{c_k\boldsymbol{\Lambda}_k}f)(\hat{\boldsymbol{\mu}}_k) - (\mathscr{D}^2_{-c_k\boldsymbol{\Lambda}_k}f)(\hat{\boldsymbol{\mu}}_k-tc_k\boldsymbol{\Lambda}_k) + (\mathscr{D}^2_{-c_k\boldsymbol{\Lambda}_k}f)(\hat{\boldsymbol{\mu}}_k)\Big]\mathrm{d}t\right| |\hat{\boldsymbol{\mu}}_k\right).$$

When we expand the differential operators, we can see that $(\mathscr{D}^2_{c_k\boldsymbol{\Lambda}_k}f)(\hat{\boldsymbol{\mu}}_k) = (\mathscr{D}^2_{-c_k\boldsymbol{\Lambda}_k}f)(\hat{\boldsymbol{\mu}}_k)$ and that $(\mathscr{D}^2_{-c_k\boldsymbol{\Lambda}_k}f)(\hat{\boldsymbol{\mu}}_k - tc_k\boldsymbol{\Lambda}_k) = (\mathscr{D}^2_{c_k\boldsymbol{\Lambda}_k}f)(\hat{\boldsymbol{\mu}}_k - tc_k\boldsymbol{\Lambda}_k)$. Therefore,

$$\begin{aligned}|b_{k,l}(\hat{\boldsymbol{\mu}}_k)| &\leq \mathbb{E}\left(\left|\int_0^1 \frac{1-t}{2c_k\Delta_{k,l}}\Big[(\mathscr{D}^2_{c_k\boldsymbol{\Lambda}_k}f)(\hat{\boldsymbol{\mu}}_k+tc_k\boldsymbol{\Lambda}_k) + (\mathscr{D}^2_{c_k\boldsymbol{\Lambda}_k}f)(\hat{\boldsymbol{\mu}}_k-tc_k\boldsymbol{\Lambda}_k)\Big]\mathrm{d}t\right| |\hat{\boldsymbol{\mu}}_k\right)\\ &\leq \mathbb{E}\left(\int_0^1 \frac{1-t}{2c_k|\Delta_{k,l}|}\Big[|(\mathscr{D}^2_{c_k\boldsymbol{\Lambda}_k}f)(\hat{\boldsymbol{\mu}}_k+tc_k\boldsymbol{\Lambda}_k)| + |(\mathscr{D}^2_{c_k\boldsymbol{\Lambda}_k}f)(\hat{\boldsymbol{\mu}}_k-tc_k\boldsymbol{\Lambda}_k)|\Big]\mathrm{d}t\middle|\hat{\boldsymbol{\mu}}_k\right).\end{aligned}$$

Each differential operator consists of a sum over $4n^2$ terms, each the product of two components of $c_k\boldsymbol{\Lambda}_k$ and a second order derivative. From hypothesis, we have that those quantities are bounded by $|\Delta_{k,i}| \leq \alpha_0$ y $|\partial_i\partial_j f(\boldsymbol{z})| \leq \alpha_2$, so

$$|b_{k,l}(\hat{\boldsymbol{\mu}}_k)| \leq \int_0^1 \frac{1-t}{2c_k}\mathbb{E}|\Delta_{k,l}^{-1}|[8n^2c_k^2\alpha_0^2\alpha_2]\mathrm{d}t. \tag{15}$$

Considering that $\mathbb{E}|\Delta_{k,i}^{-1}| \leq \alpha_1$,

$$|b_{k,l}(\hat{\boldsymbol{\mu}}_k)| \leq 2c_kn^2\alpha_0^2\alpha_1\alpha_2. \tag{16}$$

□

Theorem 2. *Let the conditions of theorem 1 and the following assumptions hold:*

(i): $a_k, c_k > 0\ \forall k$; $\lim_{k\to\infty}(|a_k|+|c_k|) = 0$; $\sum_{k=1}^{\infty} a_k = \infty$; $\sum_{k=1}^{\infty}(a_k/c_k)^2 < \infty$,

(ii): *For some* $\beta_0, \beta_1, \beta_2 > 0$, $\forall k$, $\mathbb{E}(\varepsilon_{k,\pm}^2) \leq \beta_0$, $\mathbb{E}([f(\hat{\boldsymbol{\mu}}_k \pm c_k\boldsymbol{\Lambda}_k)]^2) \leq \beta_1$, *and* $\mathbb{E}(\Delta_{k,i}^{-2}) \leq \beta_2$,

(iii): $\|\hat{\boldsymbol{\mu}}_k\| < \infty$ *for almost all k,*

(iv): $\boldsymbol{z}(t) = \tilde{\boldsymbol{z}}$ *is an asymptotically stable solution of the differential equation* $\mathrm{d}\boldsymbol{z}(t)/\mathrm{d}t = -\boldsymbol{g}(\boldsymbol{\mu})$,

(v): *Consider the domain of attraction* $D(\tilde{\boldsymbol{z}}) = \{\boldsymbol{z}_0 : \lim_{t\to\infty}\boldsymbol{z}(t|\boldsymbol{z}_0) = \tilde{\boldsymbol{z}}\}$ *where* $\boldsymbol{z}(t|\boldsymbol{z}_0)$ *denotes the solutions to the differential equation of Å4 based on initial conditions* $\boldsymbol{z}(0) = \boldsymbol{z}_0$. *There exists a closed set* $S \subseteq D(\tilde{\boldsymbol{z}})$ *such that* $\hat{\boldsymbol{z}}_k \in S$ *infinitely often for almost all sample points,*

Then

$$\lim_{k\to\infty}\hat{\boldsymbol{z}}_k = \tilde{\boldsymbol{z}} \tag{17}$$

for almost all $\omega \in \Omega$.

Proof. According to [3], SPSA converges to the solution of the optimisation problem if

1. $\|\boldsymbol{b}_k(\hat{\boldsymbol{x}}_k)\| < \infty$ and $\lim_{k\to\infty}\boldsymbol{b}_k(\hat{\boldsymbol{x}}_k) = 0$ almost surely
2. $\lim_{k\to\infty}P\left(\sup_{m\geq k}\|\sum_{i=k}^{m}a_i\boldsymbol{e}_i(\hat{\boldsymbol{x}}_i)\| \geq \eta\right) = 0$ for all $\eta > 0$

Here, $\hat{\boldsymbol{x}}_k$ is the estimate at the kth iteration of the vector $\tilde{\boldsymbol{x}}$ which minimises the function $f(\boldsymbol{x})$, just like $\hat{\boldsymbol{\mu}}_k$ and $\tilde{\boldsymbol{\mu}}$ for $f(\boldsymbol{\mu})$, only for real numbers. The functions $b_k(\hat{\boldsymbol{x}}_k)$ and $e_k(\hat{\boldsymbol{x}}_k)$ are definied in a similar way, and assumptions similar to those in theorem 2 are made.

We will show that similar statements for complex numbers imply the above, and thus the convergence of SPSA, and that in turn implies the convergence of CSPSA.

Firstly, we will prove that the following hold:

1. $\|\boldsymbol{b}_k(\hat{\boldsymbol{\mu}}_k)\| < \infty$ and $\lim_{k\to\infty}\boldsymbol{b}_k(\hat{\boldsymbol{\mu}}_k) = 0$ almost surely
2. $\lim_{k\to\infty}P\left(\sup_{m\geq k}\|\sum_{i=k}^{m}a_i\boldsymbol{e}_i(\hat{\boldsymbol{\mu}}_i)\| \geq \eta\right) = 0$ for all $\eta > 0$

It is shown in theorem 1 that $|b_k(\hat{\boldsymbol{\mu}}_{k,l})|$ has an upper bound directly proportional to c_k. Due to condition (i), $\lim_{k\to\infty}c_k = 0$. This proves condition 1.

To prove condition 2, we notice that the sequence $\{\sum_{i=k}^{m}a_i\boldsymbol{e}_i(\hat{\boldsymbol{\mu}}_i)\}_{m\geq k}$ is a martingale. Indeed,

$$\mathbb{E}\left(\sum_{i=k}^{m+1} a_i \boldsymbol{e}_i(\hat{\boldsymbol{\mu}}_i) - \sum_{i=k}^{m} a_i \boldsymbol{e}_i(\hat{\boldsymbol{\mu}}_i) \middle| \hat{\boldsymbol{\mu}}_m\right) = \mathbb{E}(a_{m+1}\boldsymbol{e}_{m+1}(\hat{\boldsymbol{\mu}}_{m+1})|\hat{\boldsymbol{\mu}}_m) = a_{m+1}(\mathbb{E}(\hat{\boldsymbol{g}}_{m+1}(\hat{\boldsymbol{\mu}}_{m+1})|\hat{\boldsymbol{\mu}}_m) - \mathbb{E}(\mathbb{E}(\hat{\boldsymbol{g}}_{m+1}(\hat{\boldsymbol{\mu}}_{m+1})|\hat{\boldsymbol{\mu}}_{m+1})|\hat{\boldsymbol{\mu}}_m)).$$

But $\mathbb{E}(\mathbb{E}(\hat{\boldsymbol{g}}_{m+1}(\hat{\boldsymbol{\mu}}_{m+1})|\hat{\boldsymbol{\mu}}_{m+1})|\hat{\boldsymbol{\mu}}_m)) = \mathbb{E}(\hat{\boldsymbol{g}}_{m+1}(\hat{\boldsymbol{\mu}}_{m+1})|\hat{\boldsymbol{\mu}}_{m+1})$ because $\mathscr{F}_m \subset \mathscr{F}_{m+1}$, so

$$\mathbb{E}\left(\sum_{i=k}^{m+1} a_i \boldsymbol{e}_i(\hat{\boldsymbol{\mu}}_i) - \sum_{i=k}^{m} a_i \boldsymbol{e}_i(\hat{\boldsymbol{\mu}}_i) \middle| \hat{\boldsymbol{\mu}}_m\right) = 0. \quad (18)$$

Therefore, $\{\|\sum_{i=k}^{m} a_i \boldsymbol{e}_i(\hat{\boldsymbol{\mu}}_i)\|\}_{m\geq k}$ is a submartingale. By Doob's inequality, if we have a submartingale $\{X_n\}$, for all $\eta > 0$ and $p \geq 1$ we have

$$P\left(\max_{1\leq i\geq n} X_i \geq \eta\right) \leq \eta^{-p}\mathbb{E}(|X_n|^p). \quad (19)$$

Then,

$$P\left(\sup_{m\geq k}\left\|\sum_{i=k}^{m} a_i \boldsymbol{e}_i(\hat{\boldsymbol{\mu}}_i)\right\| \geq \eta\right) \leq \eta^{-2}\mathbb{E}\left(\left\|\sum_{i=k}^{\infty} a_i \boldsymbol{e}_i(\hat{\boldsymbol{\mu}}_i)\right\|^2\right). \quad (20)$$

Because $\mathbb{E}(\boldsymbol{e}_i^T\boldsymbol{e}_j) = \mathbb{E}(\boldsymbol{e}_i^T\mathbb{E}(\boldsymbol{e}_j|\hat{\boldsymbol{z}}_i)) = 0$ when $i < j$ we have

$$P\left(\sup_{m\geq k}\left\|\sum_{i=k}^{m} a_i \boldsymbol{e}_i(\hat{\boldsymbol{\mu}}_i)\right\| \geq \eta\right) \leq \eta^{-2}\sum_{i=k}^{\infty} a_i\mathbb{E}\left(\|\boldsymbol{e}_i(\hat{\boldsymbol{\mu}}_i)\|^2\right). \quad (21)$$

We can see that

$$\begin{aligned}\mathbb{E}(|\hat{g}_{k,l}(\hat{\boldsymbol{\mu}}_k)|^2) &= \frac{1}{4}\mathbb{E}[\mathbb{E}(|(c_k\Delta_{k,l}^*)^{-1}(f(\boldsymbol{\mu}_k + c_k\boldsymbol{\Lambda}_k)\\ &\quad - f(\boldsymbol{\mu}_k - c_k\boldsymbol{\Lambda}_k) + \varepsilon_{k,+} - \varepsilon_{k,-})|^2|\boldsymbol{\mu}_k,\boldsymbol{\Lambda}_k)]\\ &= \frac{1}{4}\mathbb{E}[|c_k\Delta_{k,l}^*|^{-2}\mathbb{E}(|f(\boldsymbol{\mu}_k + c_k\boldsymbol{\Lambda}_k)\\ &\quad - f(\boldsymbol{\mu}_k - c_k\boldsymbol{\Lambda}_k) + \varepsilon_{k,+} - \varepsilon_{k,-}|^2|\boldsymbol{\mu}_k,\boldsymbol{\Lambda}_k)]\\ &\leq \frac{1}{2}\mathbb{E}[|c_k\Delta_{k,l}^*|^{-2}\mathbb{E}(|f(\boldsymbol{\mu}_k + c_k\boldsymbol{\Lambda}_k)\\ &\quad - f(\boldsymbol{\mu}_k - c_k\boldsymbol{\Lambda}_k)|^2 + |\varepsilon_{k,+} - \varepsilon_{k,-}|^2|\boldsymbol{\mu}_k,\boldsymbol{\Lambda}_k)].\end{aligned}$$

From condition (ii), $\mathbb{E}(\varepsilon_{k\pm})^2 \leq \beta_0$ and $\mathbb{E}f(\boldsymbol{\mu}_k \pm c_k\boldsymbol{\Lambda}_k)^2 \leq \beta_1$, so

$$\mathbb{E}(|\hat{g}_{k,l}(\hat{\boldsymbol{\mu}}_k)|^2) \leq 2\mathbb{E}(|c_k\Delta_{k,l}|^{-2}(\beta_0+\beta_1)). \quad (22)$$

We also have that $\mathbb{E}(|\Delta_{k,l}|^{-2}) \leq \beta_2$, thus

$$\mathbb{E}(|\hat{g}_{k,l}(\hat{\boldsymbol{\mu}}_k)|^2) \leq 2(\beta_0+\beta_1)\beta_2 c_k^{-2}. \quad (23)$$

On the other hand, we have that

$$\begin{aligned}\mathbb{E}(\|\boldsymbol{e}_k(\hat{\boldsymbol{\mu}}_k))\|^2) &= \mathbb{E}(\|\hat{\boldsymbol{g}}_k(\hat{\boldsymbol{\mu}}_k) - \mathbb{E}(\hat{\boldsymbol{g}}_k(\hat{\boldsymbol{\mu}}_k)|\hat{\boldsymbol{\mu}}_k))\|^2)\\ &\leq \mathbb{E}(\|\hat{\boldsymbol{g}}_k(\hat{\boldsymbol{\mu}}_k)\| + \|\mathbb{E}(\hat{\boldsymbol{g}}_k(\hat{\boldsymbol{\mu}}_k)|\hat{\boldsymbol{\mu}}_k)\|)^2\\ &\leq 2\mathbb{E}(\|\hat{\boldsymbol{g}}_k(\hat{\boldsymbol{\mu}}_k)\|^2) + 2\mathbb{E}(\|\mathbb{E}(\hat{\boldsymbol{g}}_k(\hat{\boldsymbol{\mu}}_k)|\hat{\boldsymbol{\mu}}_k)\|^2)\\ &= 4\sum_{l=1}^{n}\mathbb{E}(|\hat{g}_{k,l}(\hat{\boldsymbol{\mu}}_k)|^2)\\ &\leq 8n(\beta_0+\beta_1)\beta_2 c_k^{-2}.\end{aligned}$$

Substituting in (21), we obtain

$$P\left(\sup_{m\geq k}\left\|\sum_{i=k}^{m} a_i \boldsymbol{e}_i(\hat{\boldsymbol{\mu}}_i)\right\| \geq \eta\right) \leq \frac{8n}{\eta^2}(\beta_0+\beta_1)\beta_2\sum_{i=k}^{\infty}\left(\frac{a_i}{c_i}\right)^2 \quad (24)$$

Condition (i) says that $\sum_{i=0}^{\infty}\left(\frac{a_i}{c_i}\right)^2 < \infty$, so $\lim_{k\to\infty}\sum_{i=k}^{\infty}\left(\frac{a_i}{c_i}\right)^2 = 0$. Statement 2 follows.

We will show that statements 1 and 2 for CSPSA satisfy the conditions for Theorem 2.3.1 in [4]. Because we can regard any function of $\boldsymbol{\mu}$ as a function of $\boldsymbol{x}$ [1], we have that $\boldsymbol{b}_k(\hat{\boldsymbol{\mu}}_k) = \boldsymbol{b}_k(\hat{\boldsymbol{x}}_k)$ and $\boldsymbol{e}_k(\hat{\boldsymbol{\mu}}_k) = \boldsymbol{e}_k(\hat{\boldsymbol{x}}_k)$. According to [4], this implies that the algorithm

$$\hat{\boldsymbol{x}}_{k+1} = \hat{\boldsymbol{x}}_k - a_k[\boldsymbol{\nabla} f(\hat{\boldsymbol{x}}_k) + \boldsymbol{b}_k(\hat{\boldsymbol{x}}_k) + \boldsymbol{e}_k(\hat{\boldsymbol{x}}_k)] \quad (25)$$

satisfies

$$\lim_{k\to\infty}\hat{\boldsymbol{x}}_k = \tilde{\boldsymbol{x}} \quad (26)$$

with $\tilde{\boldsymbol{x}} = (\mathrm{Re}(\tilde{\boldsymbol{z}}), \mathrm{Im}(\tilde{\boldsymbol{z}}))$. Using (2), we can rewrite the CSPSA algorithm as

$$\hat{\boldsymbol{z}}_{k+1} = \hat{\boldsymbol{z}}_k - a_k[\partial_{\boldsymbol{z}^*} f(\hat{\boldsymbol{\mu}}_k) + \boldsymbol{b}_k(\hat{\boldsymbol{\mu}}_k) + \boldsymbol{e}_k(\hat{\boldsymbol{\mu}}_k)]. \quad (27)$$

Because $\partial_{\boldsymbol{z}^*} = \partial_x + i\partial_y$, we can identify algorithm (25) with (27), so $\hat{\boldsymbol{x}}_k = (\mathrm{Re}(\hat{\boldsymbol{z}}_k), \mathrm{Im}(\hat{\boldsymbol{z}}_k))$. Therefore,

$$\lim_{k\to\infty}\hat{\boldsymbol{z}}_k = \tilde{\boldsymbol{z}}. \quad (28)$$

□

[1] L. Sorber, M. van Barel, and L. de Lathauwer, SIAM J. Optim. **22**, 879 (2012).

[2] H. Amann and J. Escher, *Analysis II* (Springer Science & Business Media, Basel, 2008).

[3] J. C. Spall, IEEE Trans. Autom. Control **37**, 332 (1992).
[4] H. J. Kushner and D. S. Clark, *Stochastic approximation methods for constrained and unconstrained systems* (Springer-Verlag New York, 1978).
